# Comparison of intracytoplasmic sperm injection outcomes in azoospermic men who underwent testicular sperm extraction vs. microdissection testicular sperm extraction: A cross-sectional study

**DOI:** 10.18502/ijrm.v19i9.9716

**Published:** 2021-10-10

**Authors:** Serajoddin Vahidi, Nima Narimani, Saeid Abouei, Ali Sadeghi, Keivan Lorian, Amirhossein Rahavian

**Affiliations:** ^1^Andrology Research Center, Yazd Reproductive Sciences Institute, Shahid Sadoughi University of Medical Sciences, Yazd, Iran.; ^2^Hasheminejad Kidney Center, Iran University of Medical Sciences, Tehran, Iran.; ^3^Student Research Committee, Faculty of Medicine, Shahid Sadoughi University of Medical Sciences, Yazd, Iran.; ^4^Department of Surgical Technology, Faculty of Paramedical, Shahid Sadoughi University of Medical Sciences, Yazd, Iran.; ^5^Research and Clinical Center for Infertility, Yazd Reproductive Sciences Institute, Shahid Sadoughi University of Medical Sciences, Yazd, Iran.

**Keywords:** Intracytoplasmic sperm injection, Azoospermia, Testicular sperm extraction, Microdissection testicular sperm extraction, Pregnancy outcome.

## Abstract

**Background:**

Outcomes of intracytoplasmic sperm injection (ICSI) may be different in azoospermic men who undergo testicular sperm extraction (TESE) vs. microdissection-TESE (micro-TESE).

**Objective:**

This study was conducted to compare the ICSI outcomes in men who underwent TESE vs. micro-TESE due to obstructive azoospermia and nonobstructive azoospermia, respectively.

**Materials and Methods:**

A total of****310****azoospermic men who underwent ICSI from September 2016 to September 2020 were enrolled in this cross-sectional study and divided into two groups (172 cases in the TESE and 138 cases in the micro-TESE group). The paternal and maternal age, and the fertilization, biochemical pregnancy, abortion and live birth rates were compared between the two groups.

**Results:**

Maternal mean age was significantly higher in the TESE group (34.9 ± 4.2 yr vs. 32.3 ± 5.7 yr). The fertilization and biochemical pregnancy rates were significantly higher in the TESE group, but the abortion rate was similar in the two groups. The live birth rate was higher in the TESE group, but this difference was not significant (p = 0.06). Also, the maternal and paternal age did not affect ICSI outcomes.

**Conclusion:**

Individuals who underwent TESE had higher fertilization and biochemical pregnancy rates than those who underwent micro-TESE, but the live birth rate was not significantly different.

## 1. Introduction

The worldwide estimated prevalence of infertility is about 10-15% (1); however, primary infertility in Iran is about 25% (2). About 10-15% of infertile men are azoospermic (3). Azoospermia is defined as the complete absence of sperm in the semen sample and is categorized into two groups: obstructive (OA) and nonobstructive (NOA) (4, 5). With the advent of intracytoplasmic sperm injection (ICSI) in 1992, the azoospermic men who once were considered sterile, regained the chance to have their own biological children (6). OA is further categorized as primary (congenital) or secondary (acquired). Assisted reproductive techniques are commonly advised in cases of the primary form and when the reconstruction of the complex secondary structure is impossible. Given that there is normal spermatogenesis in the testes in OA, sperms can be retrieved through different methods (5).

Conversely, azoospermia in NOA may be due to disrupted spermatogenesis. Given the aggressiveness and low success rate of sperm retrieval by testicular sperm extraction (TESE) in NOA, Schlegel introduced microdissection sperm retrieval (micro-TESE) to improve the sperm recovery rate and testis viability preservation. The sperm retrieval rate of micro-TESE is about 16%–63% (7, 8).

Different studies have reported different fertilization, pregnancy, miscarriage and live birth rates between these two groups. For instance, one study found lower fertilization rates in men with NOA but similar clinical pregnancy rates between OA and NOA groups (5). Some studies have reported similar ICSI outcomes in men with OA vs. NOA, including a retrospective study in 2008 which found that the miscarriage rate did not significantly differ between the NOA and OA groups (9). Other studies have shown higher pregnancy rates in OA cases (10-12). Furthermore, some studies have revealed that factors like maternal age can influence the success rate of ICSI (13).

For successful in vitro fertilization, it is necessary for both the sperm and oocyte to be high quality (14, 15). Some oocyte quality criteria that can be assessed non-invasively are the dispersion cumulus cell, and the existence of a first polar body which shows nuclear maturity and no sign of atresia. Other abnormalities which can affect oocyte quality and total fertilization failure (TFF) are the accumulation of smooth endoplasmic reticulum discs and asynchronous nuclear and cytoplasmic maturation (16).

Two important parameters for the oocyte's developmental potential are mitochondrial function and intracellular Ca${}^{2+}$. The activity of the mitochondria and mitochondrial metabolism are regulated by mitochondrial Ca${}^{2+}$. Sperm-oocyte fusion activates Ca${}^{2+}$ oscillation, which plays a critical role in changing the state of the fertilized oocyte's mitochondria from its resting state to the active state. Then the mitochondrial activity maintains the Ca${}^{2+}$ oscillations (17).

So, we conducted this study to evaluate differences in the results of ICSI between OA cases who underwent TESE vs. NOA cases who underwent micro-TESE for sperm extraction.

## 2. Materials and Methods

A total of 310 azoospermic men who underwent ICSI for the first time from September 2016 to September 2020 in the Yazd Reproductive Sciences Institute, a tertiary referral center for infertility in Iran, were enrolled in this retrospective cross-sectional study. Cases were divided into two groups: one group underwent TESE for sperm extraction (n = 172) and the other group underwent micro-TESE (n = 138). The inclusion criteria for the TESE group were normal testes volume and acceptable hormonal assay (which was defined as blood luteinizing hormone (LH) and follicle stimulating hormone (FSH) levels < two times higher than the normal range). The inclusion criteria for the micro-TESE group were low testicular volume and high hormonal assay (blood LH and FSH levels ≥ two times higher than the normal range). The inclusion criterion of the female partner was the best quality of oocyte (spherical, no cytoplasmic granules, no aggregation of the smooth endoplasmic reticulum discs, no vacuoles). The included women were aged < 40 yr and the men were < 50 yr. Men with a history of bilateral vasectomy, varicocelectomy, a testicular tumor, chemo-radiation, hypogonadotropic hypogonadism, epididymo-orchitis, or genetic abnormality were excluded from the study.

Medical records of these individuals were reviewed, and the maternal and paternal age, and the fertilization, biochemical pregnancy, abortion and live birth rates were extracted from the files and compared between the groups. The effect of maternal and paternal age on ICSI outcomes was analyzed.

Biochemical pregnancy and abortion were defined as a positive human chorionic gonadotropin (hCG) test three weeks after implantation and a miscarriage at least three months after implantation, respectively. Semen analysis was performed and only azoospermia cases were selected. TFF occurred in 41% of TESE cases and 55% of micro-TESE cases.

### Ethical considerations

This study was approved by the ethics committee of Research and Clinical Center for Infertility, Shahid Sadoughi University of Medical Sciences, Yazd, Iran (Code: IR.SSU.RSI.REC.1400.006). Consent was obtained from all participants orally.

### Statistical analysis 

The Statistical Package for the Social Sciences (SPSS) version 20 (IBM, California, United States) was used to perform Chi-square tests for the qualitative data, and the quantitative outcomes were analyzed through descriptive statistics (mean ± standard deviation) and independent *t* tests. Regression analysis was performed to determine the association of maternal and paternal age with ICSI outcomes. P < 0.05 was considered the statistical significance level.

## 3. Results

Over four years, a total of 555 ICSI procedures were performed in our Center. 254 (46%) cases underwent micro-TESE for sperm extraction, and the others underwent TESE and****percutaneous epididymal sperm aspiration. 245 (44.1%) cases were excluded from the study based on the inclusion and exclusion criteria, and the remaining cases were categorized into two groups (TESE and micro-TESE). The mean paternal age was significantly different between the two groups (40.65 ± 9.22 yr vs. 36.70 ± 5.86 yr for the TESE and micro-TESE groups, respectively, with a range of 24-50 yr; p ≤ 0.001). Also, the mean age of women was significantly higher in the TESE group (34.97 ± 4.22 yr vs. 32.35 ± 5.77 yr, with a range of 18-40 yr; p ≤ 0.001). The ICSI outcomes of the two groups are shown in Table I.

Logistic regression analysis was done to evaluate the impact of maternal and paternal age on ICSI outcomes, and it revealed that there was no relationship between these variables (Table II).

**Table 1:** The ICSI outcomes of the TESE and micro-TESE groups


[1.5in,lr]**ICSI outcomes** **Groups**	**TESE (n = 172)**	**Micro-TESE (n = 138)**	**p-value**
**Positive fertilization**	131 (76.1)	83 (60.0)	≤ 0.001
**Positive biochemical pregnancy**	45 (26.1)	20 (14.4)	0.01
**Abortion**	7 (4.0)	5 (3.6)	1.00
**Live birth**	21 (12.2)	9 (6.5)	0.09
Data presented as n (%). Chi-square test was used. ICSI: Intracytoplasmic sperm injection, TESE: Testicular sperm extraction, Micro-TESE: Micro-testicular sperm extraction			

**Table 2:** Independent factors related to fertilization, biochemical pregnancy, abortion and live birth rate in individuals who underwent ICSI


**Variable**	Maternal age*	**p-value**	Paternal age*	**p-value**
**Fertilization rate**	1.01 (0.96-1.06)	0.56	0.99 (0.95-1.03)	0.62
**Biochemical pregnancy rate**	0.98 (0.92-1.04)	0.60	0.99 (0.94-1.04)	0.86
**Abortion rate**	0.98 (0.87-1.09)	0.76	1.04 (0.96-1.11)	0.33
**Live birth rate**	1.01 (0.92-1.10)	0.83	0.94 (0.86-1.02)	0.16
*Data presented as OR (95% CI). Logistic regression was used. ICSI: Intracytoplasmic sperm injection, OR: Odds ratio, CI: Confidence interval				

## 4. Discussion

In the present study, the outcomes of ICSI in OA cases who underwent TESE and NOA cases who underwent micro-TESE for sperm extraction were investigated. In total, 555 men with OA and NOA were involved in the ICSI technique and 310 of these were included in this study. The data analysis demonstrated that the individuals with OA had a 76.1% positive fertilization rate and individuals with NOA had a 60.0% fertilization rate. Moreover, the positive biochemical pregnancy rate was significantly higher in OA individuals. The live birth rate was also higher in OA individuals compared to the individuals with NOA, but the abortion rate was similar. In addition, the mean paternal age was significantly different between the two groups. Also, the mean age of the women was significantly higher in the TESE group.

NOA individuals are classified into two groups: hypogonadotropic hypogonadism and hypergonadotropic hypogonadism or eugonadism. Individuals with hypogonadotropic hypogonadism are characterized by low FSH, low LH and low testosterone levels. The congenital cause for this group is Kallmann syndrome (hypothalamic GnRH deficiency) and the acquired cause is pituitary tumors. In hypogonadotropic hypogonadism cases, gonadotropin therapy is started for three to six months to induce spermatogenesis. hCG and FSH are used. hCG is sufficient to induce spermatogenesis but FSH optimizes this process, especially in individuals with congenital abnormalities. Pulsatile GnRH injection is another therapy for this group. The hypergonadotropic hypogonadism or eugonadism group can be diagnosed by normal/high FSH, normal/high LH and normal/low testosterone levels. Genetic abnormalities (chromosomal) are the congenital cause of this group, and varicocele, orchitis, gonadotoxins (chemotherapy/radiation), trauma/torsion and idiopathic reasons are the acquired causes of these cases.

The spermatogenesis process requires high levels of intra-testicular testosterone. The aromatase enzyme converts androgens to estrogens in men. Aromatase inhibitors block this conversion and have a potential role in NOA individuals (18). Antiestrogens and gonadotropins are also used in the treatment of this group. Microsurgical reconstruction or sperm retrieval processes that are used in assisted reproductive techniques are the main treatments for men with OA (19).

Para-clinical tests such as hormone and genetic assessments help to understand the causes of azoospermia, especially NOA. Through hormone assessment, the condition can be properly diagnosed and the physician can better initiate hormone therapy. Moreover, these tests can help to predict the success of medical therapy and surgical sperm retrieval (18).

In this study, it was proposed that men with NOA who underwent ICSI would have poorer outcomes in all parameters than men with OA. According to the results, the men with NOA had a significantly lower fertilization and clinical pregnancy rate than the men with OA. The reason for the lower fertilization rate in NOA cases is the early paternal effect, which can be caused by dysregulation and dysfunction of oocyte-activating factors, centrosomes, and cytoskeletal components (20, 21).

It has been shown that miscarriage rates may be higher in men with NOA due to aneuploidy and DNA damage in NOA spermatozoa that may contribute to a late paternal effect, leading to pregnancy loss (20, 22, 23). The results of the present study are similar to those of several other studies that revealed significant higher fertilization rates in the men with OA compared with in those with NOA (11, 24, 25). These results can be explained by the sperm functional impairment in men with NOA, which increases genetic abnormalities in these men (26). However, in research conducted in 2015, no differences were observed in the fertility rate or clinical pregnancy rate between the OA and NOA groups (27). In 2005 a metaanalysis revealed that fertilization rate is lower in NOA cases (5). A retrospective study showed lower pregnancy rates in men with NOA than in men with OA (9). In another study, it was indicated that the clinical pregnancy rate in men with NOA was lower than in men with OA (28). In another comparison of NOA and OA outcomes, the implantation rate was found to be lower in the NOA group (29).

Factors that can affect the outcome of ICSI when using non-ejaculated sperm include the azoospermia etiology, the age of the female partner, and the sperm status (fresh or thawed) (30). A younger maternal age has been found to have a positive effect on the fertilization and clinical pregnancy rates of OA men (30). However, some studies have suggested that maternal age does not impact fertilization rates but only affects pregnancy rates (13, 31). Overall, both men with OA and NOA can have successful pregnancies. More research is required to understand the impact of variables such as maternal age and paternal effect on the fertilization rate and miscarriage in men with OA and NOA. Finding and understanding more about factors that affect ICSI outcomes can help us to predict the outcome of fertility procedures and advise our cases better.

## 5. Conclusion

This study confirmed previous findings that men with OA had significantly higher fertilization and biochemical pregnancy rates than men with NOA. The live birth rate was higher in men with OA, but this difference was not significant.

##  Conflict of Interest

There is no conflict of interest.
